# Quality of life among adults with less severe malocclusions seeking orthodontic treatment

**DOI:** 10.2340/aos.v84.43536

**Published:** 2025-05-13

**Authors:** Narmina Sandlund, Idil Burt, Robert Schibbye

**Affiliations:** a Distriktstandvården Sverige, Stockholm, Sweden; b Folktandvården Eastmaninstitutet Ortodonti, Stockholm, Sweden; c Department of Dental Medicine, Karolinska Institutet, Huddinge, Sweden

**Keywords:** orthodontics, malocclusion, oral health, quality of life, body dysmorphic disorders

## Abstract

**Objective:**

Severe malocclusions affect Oral Health Related Quality of Life (OHRQoL), but the effects of less severe malocclusions are underexplored. This study investigates OHRQoL and psychological well-being in adults with less severe malocclusion, but high subjective need of orthodontic treatment.

**Material and methods:**

This cross-sectional study included 130 study participants aged 18–75 years, with less severe malocclusion. Two groups were assessed: 65 with high subjective orthodontic treatment need and 65 in a control group. Patients with malocclusions graded with Index of Orthodontic Treatment Need–Dental Health Component (IOTN-DHC) index 1, 2, or 3 were included. Three questionnaires were used: Oral Health Impact Profile (OHIP-14), Hospital Anxiety and Depression Scale (HADS), and a general appearance perception survey.

**Results:**

The high subjective need group showed significantly higher OHIP-14 scores, reflecting poorer OHRQoL, compared to the control group. When age was considered, only the orofacial appearance subscale remained significant. No clinical signs of depression or anxiety were found among participants. However, a subset in the high subjective need group spent over an hour daily on their oral appearance.

**Conclusions:**

Adults seeking orthodontic treatment despite low objective treatment need may have impacted OHRQoL and an impairment from the time spent on their oral appearance. Dentist should consider patients’ subjective treatment needs and impact on daily functioning when treating patients with mild malocclusions.

## Introduction

There has been a notable surge in orthodontic treatments among adults in recent years. The growing interest in aligner systems highlights the rising demand for aesthetic dental corrections among adults [1, 2]. Earlier research has established a correlation between malocclusion and perceived Oral health Related Quality of Life (OHRQoL) [3–6], demonstrating that more severe malocclusions significantly impact an individual’s quality of life [4, 7–9]. However, many studies focus on patients with high objective need, leaving a gap in knowledge about how OHRQoL is impacted in patients with low objective need. An individual’s satisfaction with their dental aesthetics may be inconsistent with the standardised methods of measurements for malocclusion and misalignments. It appears that some people have a great concern about minor misalignments, while others do not show any concerns with having severe aesthetical issues [10]. Cosmetic dental procedures affect both a patients appearance and their psychological well-being, self-esteem, body image, and confidence [11]. As orthodontic treatments can also be viewed as cosmetic procedures, it is crucial to consider not only physical, but also psychological effects, particularly in patients with less severe malocclusions seeking treatments mainly for cosmetic reasons.

Body Dysmorphic Disorder (BDD), a psychiatric diagnosis resulting in an increased preoccupation about a minor or perceived defect of an individual’s physical appearance, can play a role in self-perception [12] and might therefore, also affect their self-perceived malocclusion. Patients with BDD can perceive minor abnormalities as exaggerated, which results in a negative effect on the patient’s life and functioning. Individuals who allocate more than 1 hour per day to their perceived flawed appearance may display symptoms associated with BDD [13]. Prevalence of BDD among orthodontic patients has been estimated to be 2.8–7.5% [14, 15], but research on the prevalence specifically in patients with less severe malocclusions and high subjective need of orthodontic treatment remains limited. Identifying patients with BDD-symptoms may be crucial for orthodontists who face challenges when treating patients with incongruous expectation, as they often exhibit dissatisfaction with aesthetic dental treatments, including orthodontic procedures [15].

There is a gap in understanding how adult patients with less severe malocclusions, but with high subjective orthodontic treatment need perceive their OHRQoL and whether it is connected to aspects of their psychological well-being. Furthermore, the majority of available research observes a child population with severe malocclusions and no previous studies has explored how subjective orthodontic treatment need of patients affect their psychological well-being. Therefore, the aim of this study is to investigate the OHRQoL and the psychological well-being in adult patients with less severe malocclusion, but high subjective orthodontic treatment need.

## Material and methods

### Subjects

The study comprised 130 participants consisting of adult patients above 18 years of age attending Specialistkliniken in Kungsholmen (Specialist Dental Clinic) and Distriktstandvården in Sundbyberg (General Practice) in Stockholm, Sweden.

In Sweden, there is systemised selection of children and young adults below 23 years of age with orthodontic treatment need (malocclusions assessed as Index of Orthodontic Treatment Need–Dental Health Component [IOTN-DHC] [16] index 4 and 5) who are eligible for subsidised treatment. Various regions in Sweden employ different indices to assess malocclusions and establish specific criteria governing the qualifications for subsidised treatment. Adults over 23 years of age are eligible for subsidised orthodontic treatments only if diagnosed with severe malocclusions. Consequently, we defined our study group as adult patients with high subjective orthodontic treatment need who sought orthodontic treatment without any financial subsidies, thus covering the entire cost themselves. Swedish-speaking patients who had commenced orthodontic treatment at the two above-stated clinics between the dates of October 2022 until December 2023 were invited to participate in the study.

To be able to represent a general sample of participants from a general dental clinic, the control group consisted of adult patients who visited the general dental practice for other treatments than orthodontics. All participants in the control group were consecutively recruited from the waiting room at Distriktstandvården in Sundbyberg. The orthodontic treatment history of participants in the control group was not taken into account during the recruitment. However, before inclusion to the study, they were asked whether they had any subjective need for orthodontic treatment. The collection of surveys for the control group was performed during December 2023 until March 2024.

The inclusion criteria for the high subjective need group were: above age of 18 years, permanent dentition with minimum teeth six to six fully erupted, and low objective orthodontic treatment need according to IOTN-DHC [16] grade 1, 2, and 3.

Exclusion criteria was IOTN-DHC grades 4 and 5 and non-Swedish speaking patients.

### Study design

All participants were given comprehensive oral and written information about the study at their initial visit to the clinic. They signed a study participation approval form of informed consent for the study and were then given three questionnaires. The anonymised complete questionnaires were sealed in envelopes and stored securely in locked cabinet. By using the journal system Opus Dental [17] and Invisalign Doctors Site [18], the malocclusions of the participants were assessed using the IOTN-DHC index.

### Questionnaires

The following questionnaires were used for the study:

1. The Oral Health Impact Profile (OHIP-14) (Swedish version) [19] for the rating of participants OHRQoL. The questionnaire consists of 14 statements where participants will rate how frequent they experience the statement. The answers contain a 5-point Likert-scale from ‘never’ to ‘very often’, and answers are valued according to a point scale between 0 and 4 (0 – never, 1 – hardly ever, 2 – occasionally, 3 – fairly often, 4 – very often and ‘not applicable’ – if they cannot/do not wish to answer, with a total score from 0 to 56, a high score indicates a negative perceived OHRQoL.
2. Hospital Anxiety and Depression Scale (HADS) (Swedish version) [20], a self- report questionnaire measuring symptoms of depression and anxiety [21]. The questionnaire consists of 14 statements with 7 statements for each subscale (HADS-*anxiety* and HADS-*depression*), where participants will rate how frequent they experience a given statement. The answers contain a 4-point Likert-scale with statements consisting of answers such as ‘often’, ‘usually’, ‘occasionally’, ‘almost never’ etc. The answers are valued according to a Likert point scale between 0 and 3 with a total score of 21 points, where a total point <7 indicates depression or anxiety, 8–10 indicates possible depression or anxiety, >11 likely have depression or anxiety, and 15–21 is an indication for severe depression or anxiety [21].
3. Additionally, we created a questionnaire with four questions in relation to the patients’ general perception of their aesthetics regarding their mouth and their teeth, and one general question regarding the participants’ general perception of their mouth and teeth (Appendix 1). The four psychological questions are modified from the original BDD– Questionnaire (BDDQ), a brief self-report screening measure for BDD with high sensitivity and specificity for diagnosing BDD [13], and served as a guide to examine if the participants had any indicators consistent with BDD, namely how much time they spend on and are affected by their oral appearance in different ways. The modification of the questions from the original BDDQ was made by a clinical psychologist working within the dental field, and questions were modified to enhance their applicability when asking participants regarding the appearance of their teeth and mouth. The last fifth question consists of self-assessment of participants’ general satisfaction of the appearance of their mouth and teeth, using a 10-point scale, where 1 is not satisfied and 10 is very satisfied.

### Interrater reliability

Cohen’s kappa coefficient was used to assess the interrater reliability between the grading of malocclusion with the IOTN-DHC index. Two assessors (including a general dentist and a specialist in orthodontics) were selected for assessment of the malocclusions of 10 randomly selected patients from the sample. Patient’s photos and digital models were assessed by using Invisalign Doctor Site digital model system [18]. The interrater reliability of the 10 randomly selected patients was calculated with the Cohen kappa formula to 0.81, a strong agreement.

### Statistical analysis

The statistical analysis was done using the software IBM Corp. Released 2021. IBM SPSS Statistics for Windows, Version 28.0. Armonk, NY: IBM Corp. All data were analysed once all 130 participants were recruited. For evaluation of the questionnaires, descriptive statistics were used, where frequencies and mean values were calculated. Likert scale answers from questionnaires 1 and 2 were analysed as interval data, and therefore presented with means and standard deviations. To check the normality of the data, the Shapiro-Wilk test was used in SPSS which presented a significant value less than 0.05, indicating a non-normal distribution. Mann-Whitney U test was therefore used for OHIP-14 and HADS questionnaires for comparison between the groups. Spearman’s correlation test was used to evaluate the relationship between continuous/ordinal data, and between OHIP-14 and HADS questionnaires. Pearson’s Chi-square test, Fisher’s Exact test (only in Appendix 2, Table 3), and Fisher-Freeman-Halton Exact test were used for categorical data for questionnaire of perceived appearance of teeth/mouth. Quade Non-Parametric Analysis of Covariance was performed for controlling the effects of covariates in the analysis.

To obtain a power of 80% with a significance level of α = 0.05, with an expected effect size set of Cohen *d* = 0.7 [22] sample size of this study was calculated to consist of a minimum of 35 participants in each group. However, since our analysis also aimed to identify participants with signs of BDD, and previous studies have reported a prevalence of BDD in orthodontic patients ranging between 2.8 and 7.5% [14, 15], we increased the sample size further to enhance the likelihood of detecting also these cases.

### Ethical applications

The study was approved by Etikprövningsmyndigheten (Swedish Ethical Review Authority) in Stockholm County, Sweden (diarie nr: 2022-06522-01).

## Results

### Study sample

Participants in the high subjective need group had a statistically significant (*p* < 0.001) lower mean age (min. 18 years, max. 62 years) compared to those in the control group (min. 18 years, max 75 years), see Table 1. Younger participants in the high subjective need group also reported higher total OHIP-scores (*p* = 0.001). For the control group, there was a trend towards a younger age and higher OHIP-14 scores, but this trend was not statistically significant.

**Table 1:** Descriptive data for study participants.

Characteristic	High subjective need group (*n* = 65)			Control group (*n* = 65)		
	Mean	SD	*n*/total (%)	Mean	SD	*n*/total (%)
Age	31.9	9.8		43.2	15.1	
Gender						
Female			41/65 (63%)			35/65 (54%)
Male			24/65 (37%)			30/65 (46%)
Distribution of malocclusion						
IOTN-DHC 1			3/65 (5%)			7/65 (11%)
IOTN-DHC 2			13/65 (20%)			23/65 (35%)
IOTN-DHC 3			49/65 (75%)			35/65 (54%)

IOTN-DHC: Index of Orthodontic Treatment Need–Dental Health Component.

Gender distribution across both groups had more female participants, with no statistical significance in gender distribution between the groups. A sub-analysis showed that in control group, male participants showed higher depression scores (*p* < 0.022) and spent more time on thinking about the appearance of their teeth and mouth (*p* < 0.007) than female participants. No other statistically significant differences in questionnaire responses were found between female and male participants in any of the groups.

Most malocclusions consisted of IOTN-DHC grade 3. The high subjective need group had proportion of IOTN-DHC 3 malocclusions, accounting for 75% of cases, compared to 54% in the control group (*p* = 0.035). There were no statistically significant differences observed among responses across different IOTN-indices in either group.

### OHIP-14 and HADS scores

Table 2 shows the results from the OHIP-14 and HADS scores in the different groups. Participants within the high subjective need group reported higher total OHIP-14 scores (*p* = 0.05) in comparison to those in the control group, meaning that their perceived OHRQoL was lower. After adjusting for age as a covariate in our analysis – due to the observed age difference between the groups – the difference in total OHIP-score became non-significant (*p* = 0.161), indicating that there were no differences between total OHIP-scores between the groups when controlling for age. The orofacial appearance (*p* < 0.01) and psychosocial impact (*p* = 0.043) sub-scales demonstrated statistically significant differences, with a lower OHRQoL observed in the high subjective need group compared to the control groups. After adjusting for age as a covariate, the correlation of orofacial appearance remained statistically significant (*p* > 0.001), but the total psychosocial impact between the groups became non-significant (*p* = 0.590).

**Table 2:** OHIP-14 and HADS scores for study participants.

Variable	High subjective need group (*n* = 65)					Control group (*n* = 65)					*P* *	*P* **
	Median	Mean	SD	Min	Max	Median	Mean	SD	Min	Max		
OHRQoL (OHIP-14)												
Oral Function	1	1.7	2.1	0	8	1	1.8	1.9	0	7	0.428	0.293
Orofacial Pain	0	0.6	0.9	0	3	0	0.5	0.9	0	4	0.898	0.492
Orofacial Appearance	1	1.7	1.3	0	4	0	0.5	0.9	0	4	**<0.001**	**<0.001**
Psychosocial Impact	4	4.8	4.2	0	15	2	3.2	3.3	0	14	**0.043**	0.590
Total score	10	11	8.9	0	34	5	6.5	5.9	0	21	**0.005**	0.161
HADS-14												
Anxiety	5	4.8	2.7	0	12	4.0	3.9	3.0	0	13	**0.048**	0.184
Depression	2	2.3	1.8	0	7	1.5	2.3	2.3	0	9	0.476	0.572
Total score	6	7.2	3.9	0	17	6.0	6.2	4.7	0	18	0.143	0.296

Scores of OHIP-14 (for OHRQoL) and HADS-14 (anxiety and depression) for high subjective need group and control group. Higher OHIP-14 scores indicate lower OHRQoL and higher HADS-14 scores indicates higher anxiety and depression. OHRQoL: Oral Health Related Quality of Life; OHIP: Oral Health Impact Profile; HADS: Hospital Anxiety and Depression Scale.

* All tests were made using Mann-Whitney U test.

** Quade Non-Parametric Analysis of Covariance for controlling the effects of age as covariance.

**Table 3:** Descriptive data of participants’ perception of their oral appearance in frequencies and percentages.

Question	High subjective need group (*n* = 65)	Control group (*n* = 65)	*P*
	*n*/total (%)	*n*/total (%)	
1. Avoided things because of the appearance of your teeth/mouth			<0.001*
No	34/65 (52%)	65/65 (100%)	
Yes	31/65 (48%)	0/65 (0%)	
2. Hours/day spent on thinking about the appearance of your teeth/mouth			<0.001**
Less than every day	30/65 (46%)	59/65 (91%)	
Under 1 hour/day	28/65 (43%)	5/65 (8%)	
Between 1 and 3 hours/day	6/65 (9%)	1/65 (1%)	
Over 3 hours/day	1/65 (2%)	0/65 (0%)	
3. Have you done things to hide the appearance of your teeth/mouth?			<0.001**
No	35/65 (54%)	62/65 (95%)	
Yes	30/65 (46%)	3/65 (5%)	
If yes, how many hours/day spend on hiding the appearance of your teeth/mouth?			
Less than every day	15/30 (50%)	3/3 (100%)	
Under 1 hour/day	11/30 (37%)		
Between 1 and 3 hours/day	1/30 (3%)		
Over 3 hours/day	3/30 (10%)		
4. Is there anything else than your teeth/mouth you are dissatisfied with?			0.004*
No	39/65 (60%)	54/65 (83%)	
Yes	26/65 (40%)	11/65 (17%)	
5. ‘On a scale from 1 to 10, how satisfied are you with the appearance of your teeth and mouth in general?			<0.001***
	4.71 (Mean)	7.42 (Mean)	

An overview of participants’ answers for Questionnaire 3. Descriptive answers to follow-up questions are described in Results section ‘Perception of oral appearance’. For question 5, a higher mean score indicates more satisfaction with the appearance of teeth/mouth.

* Pearson’s Chi-square test.

** Fisher-Freeman-Halton Exact test.

*** Mann-Whitney U test.

Both the high subjective need and control groups reported low HADS scores, not indicating strong anxiety or depression for any of the groups. However, participants in the high subjective need group showed statistically significant (*p* = 0.048) higher scores for anxiety compared to those in the control group; however when controlling for age, this was not significant (*p* = 0.184).

### Perception of oral appearance

Several significantly statistical differences were observed between the groups in their perception of and the time spent on their oral appearance (see Table 3). The high subjective need group showed a larger psychological impact on all questions. No patients in the control group reported avoiding things due to the appearance of their teeth/mouth (question 1). In the high subjective need group, 48% of participants (31 out of 65) reported avoiding certain activities for this reason. The most avoided activity was smiling (22 of 65 participants, 34%), followed by being photographed (2 of 65 participants, 3%), dating (one of 65 participants, 1.5 %), and attending at events (one of 65 participants, 1.5 %). Behaviours to hide the appearance of teeth/mouth for both groups (question 3), consisted of avoiding laughing/smiling with their mouth closed (25 of 130 participants, 19%), having treatments that included dental aesthetics (having veneers, aesthetic fillings, previous Invisalign treatments, tooth whitening) (7 of 130 participants, 5%), and hiding the mouth while eating (1 of 130 participants, 0.8%). As follow up question 4, if there is anything else than participants’ teeth/mouth that they are dissatisfied with, following issues were mentioned: displeasure about hair (4 of 130 participants, 3%), dermatological problems such as acne/rosacea (4 of 130 participants, 3%), dissatisfaction with their chin/jawline (3 of 130 participants, 2%), nose (2 of 130 participants, 1.5%), body in general (1 of 130 participants, 1%), and concerns about being overweight (1 of 130 participants, 1%).

To highlight potential signs of BDD among participants, we conducted an independent analysis of the subset within the high subjective need group who reported dedicating between 1 and 3 hours or more per day on thinking about the appearance of their teeth/mouth (see Appendix 2). This subgroup, composed exclusively of seven female participants, which was a statistically significant gender difference (*p* = 0.033). No statistical differences were found between this subgroup and the remaining high subjective need group, except for the time spent and gender. Although these analyses lacked the statistical power to detect differences based on our power analysis, we still provide this table in Appendix 2, as the data have not been previously reported in the literature.

## Discussion

### Main findings

Patients exhibiting a high subjective orthodontic treatment need, despite having a low objective need, demonstrated lower OHRQoL compared to the control group, specifically in the orofacial appearance and psychosocial impact sub-scales. However, age appears to play an important role. When age was included as a covariate in our analysis, only the orofacial appearance sub-scale remained statistically significant. Both groups displayed non-clinically normal levels of depression and anxiety [21], indicating no general effect of a high subjective orthodontic treatment need on mood or psychological well-being. However, the group with the high subjective need of orthodontic treatment was less satisfied with their oral appearance and experienced greater daily functioning impacts, spending more time avoiding, thinking about or hiding their teeth or oral appearance, compared to the control group.

### OHRQoL and psychological well-being

No previous studies have specifically examined the aspects of depression and anxiety in orthodontic patients with a high subjective need for treatment. Our findings suggest that the primary concern for this population is their perceived oral health, and that they as a group do not exhibit clinical levels of anxiety or depression. In the study, participants in the study group were recruited between October 2022 and December 2023, while recruitment for the control group took place between December 2023 and March 2024. Given this timeline, the recruitment period may have influenced participants’ anxiety or depression responses, particularly considering the potential lingering effects of the COVID-19 pandemic. However, our analysis revealed no significant difference in depression levels between the groups. Furthermore, after adjusting for age, the statistical significance for anxiety diminished, suggesting that age may be a more influential factor than recruitment period.

The most affected aspects of OHRQoL for high subjective need group were oral appearance and psychosocial impact, which reflected their daily behaviours concerning their oral appearance. Earlier studies have shown that psychosocial impact is the primary affected domain of OHRQoL in patients with severe malocclusion [23, 24], and our findings suggest that this is also true for patients seeking orthodontic treatment with less severe malocclusions.

Our analysis also shows that age plays a role in how OHRQoL correlates with perceived malocclusion. Younger individuals may be more concerned with the aesthetic and social implications of malocclusion, while older population tend to place less emphasis on these factors. This highlights the importance of considering age when evaluating the effects of malocclusion. Additionally, our populations’ mean total OHIP-14 score (mean 11) aligns with reported scores for participants with ‘borderline need’/IOTN DHC 3 (mean 9.06) and ‘treatment need’/IOTN DHC 4 and 5 (mean 12.75) [23]. However, our control group exhibited notably higher total OHIP-14 scores compared to an adult population not undergoing orthodontic treatment (mean 3.63) [7], signalling a lower perceived OHRQoL in our sample. These discrepancies may result from the fact that our participants were recruited from a waiting room in general dental practice, where dental concerns beyond orthodontics could influence perceived OHRQoL. Future studies should explore whether OHIP-14 scores differ in a population not receiving any dental treatment, and this should be considered when selecting an appropriate control group.

Traditional orthodontic treatment is usually based on focussing on correcting functional issues, based on different indices that assess the severity of the malocclusion. This leaves patients with less severe malocclusions untreated. A dentist should therefore not only assess the actual functional problems perceived in patients, but also the perceived need for treatment and discomfort from physical appearance and its impact on daily functioning. Moreover, future research should explore if patients with a high subjective need, but low objective need who undergo orthodontic treatment experience an enhanced OHRQoL and are less affected by their perception of their oral appearance when their treatment is completed.

### Perception of oral appearance

Patients in the high subjective need group experienced a greater impact on their daily lives due to concerns about the appearance of their teeth and mouth. Notably, seven individuals in this group reported spending more than 1 hour a day thinking about their malocclusion, which could contribute to an elevated perception of needing orthodontic treatment. However, this time spend could also be indicative of a possible BDD since a main feature of the diagnosis is the impairment of functioning from the amount of time spent on a perceived physical defect [13]. Previous studies report the prevalence of BDD in orthodontic patients as 2.86–7.5% [14, 15], indicating that a number of patients seeking orthodontic treatment could have BDD or display BDD symptoms. Among the high subjective need group in our study, seven participants (11%) showed symptoms consistent with BDD, based on spending over 1 hour daily on thinking about their teeth/mouth. It is essential to emphasise that the questionnaire that is used in this study was not explicitly devised for diagnosing BDD or that was the intention of this study; therefore, drawing any conclusions regarding the prevalence of BDD within this cohort should clearly not be done. However, this finding warrants further research, especially considering that individuals with BDD often fail to achieve complete satisfaction following cosmetic procedures [13]. Clinicians should be cautious when considering orthodontic treatment for such patients, making this an area for future investigations. Future research is crucial for finding ways to reliably detect signs of BDD in orthodontic patients, enabling clinicians to recognise affected patients before initiating orthodontic treatment.

Another interesting finding in our study is that all participants in the subgroup spending over an hour per day on their appearance were females. Previous studies have reported varying outcomes regarding prevalence of BDD across genders in orthodontic patients. For example, an Indian study [25] found a higher prevalence of BDD in males, whereas a study conducted in Iran [15] reported stronger correlation of BDD among females. Different outcomes may be attributed to factors such as cultural variations and perceptions of beauty standards and body image perceptions, where pressure to meet certain aesthetic standards may differ across genders and cultures.

### Strengths and limitations

We did not have enough power to detect differences in our comparison between the seven participants who spend over an hour a day and the rest of the sample. Nonetheless, we included these results for interested parties as this data has not been previously reported. We believe this group represents an important area for future research. To obtain more robust findings, future studies should employ larger sample sizes and utilise more valid methods for assessing BDD among orthodontic patients, namely clinical diagnosis made by psychologists or psychiatrists.

The control group was not matched to the study group, primarily due to a higher average age in the control group, which may introduce potential source of bias. However, because of the recruitment method used, precise matching was not feasible in this study. The age difference between the study and control groups may have influenced the overall OHIP-14 scores, and we accounted for this in our analyses by adjusting for the age-related differences where possible. Nevertheless, a matched control group would have better addressed this issue and provided a more accurate assessment of the impact of OHRQoL in comparable groups. As age has been shown to significantly influence OHRQoL outcomes, future studies should prioritise recruiting age-matched controls to facilitate more reliable comparisons.

This study addresses a previously underrepresented segment of the population. While earlier studies have emphasised the influence of psychosocial well-being on perceived OHRQoL, there is a gap in research focussing on the psychological well-being of individuals actively seeking orthodontic treatment but with low objective treatment needs. Our study fills that gap and highlights potential indicators of BDD symptoms, which could possibly affect some orthodontic patients with a high subjective need, but less objective need for treatment.

## Conclusion

The perceived oral health related quality in adult patients seeking orthodontic treatment, despite a low objective orthodontic treatment need, is lower than of a normal patient group, particularly concerning the impact of their perceived orofacial appearance. Additionally, age plays a significant role in influencing their perceived OHRQoL. However, the general anxiety and depression levels in these patients, remain within the normal range. Finally, a subgroup of the patients with a high subjective need are spending a considerable time on their oral appearance which could be seen as an impact on functioning. Dentist should ask about and evaluate a patients’ subjective need, the impact on the perceived oral health, and time spent on their oral appearance when addressing orthodontic patients with low objective treatment need.
